# Polymorphisms of *MFGE8* are associated with susceptibility and clinical manifestations through gene expression modulation in Koreans with systemic lupus erythematosus

**DOI:** 10.1038/s41598-019-55061-6

**Published:** 2019-12-06

**Authors:** Wook-Young Baek, Ji-Min Woo, Hyoun-Ah Kim, Ju-Yang Jung, Chang-Hee Suh

**Affiliations:** Department of Rheumatology, Ajou University School of Medicine, 164 Worldcup-ro, Yeongtong-gu, Suwon 16499 Korea

**Keywords:** Genetic association study, Systemic lupus erythematosus

## Abstract

Systemic lupus erythematosus (SLE) is characterized by impaired clearance of apoptotic cells. Milk fat globule epidermal growth factor 8 (MFGE8) is a protein that connects αvβ3 integrin on phagocytic macrophages with phosphatidylserine on apoptotic cells. We investigated whether genetic variation of the *MFGE8* gene and serum MFGE8 concentration are associated with SLE. Single nucleotide polymorphisms (SNPs) were genotyped and serum concentrations were analyzed. The rs2271715 C allele and rs3743388 G allele showed higher frequency in SLE than in healthy subjects (HSs). Three haplotypes were found among 4 SNPs (rs4945, rs1878327, rs2271715, and rs3743388): AACG, CGCG, and CGTC. CGCG haplotype was significantly more common in SLE than in HSs. rs4945 was associated with the erythrocyte sedimentation rate and rs1878327 was associated with alopecia, C-reactive protein, complement 3, anti-dsDNA antibody, and high disease activity. rs2271715 and rs3743388 were associated with renal disease, cumulative glucocorticoid dose, and cyclophosphamide and mycophenolate mofetil use. Serum MFGE8 concentrations were significantly higher in SLE than in HSs. Furthermore, the levels of MFGE8 were significantly higher in SLE than HSs of the rs2271715 CC genotype. In conclusion, *MFGE8* genetic polymorphisms are associated not only with susceptibility to SLE but also with disease activity through modulation of gene expression.

## Introduction

Systemic lupus erythematosus (SLE) is an autoimmune disease that affects multiple organs (skin, blood, kidney, and musculoskeletal system) that may be damaged by chronic inflammatory responses, and is characterized by the presence of various autoantibodies through loss of immunological tolerance^1^. SLE usually has highest prevalence in young women of childbearing age, and the frequency is higher in Asians than in Caucasians. When SLE occurs, the inflammatory response may cause irreversible damage, including renal failure; however, organ damage can be prevented through adequate diagnosis, assessment, and treatment. The pathogenesis of SLE is complex, and is associated with environmental and hereditary factors^2^. A family history of SLE or risk of later development is present in 10% of patients with SLE. SLE occurs with a frequency of 24% in monozygotic twins and 2% in dizygotic twins^3^. These findings imply that polymorphisms of specific genes that express complement or a major histocompatibility complex may act as hereditary factors in the development of SLE^4,5^.

Over the last decade, our understanding of the genetic basis of SLE has increased through use of genome-wide association studies (GWAS) that have provided persuasive evidence of an association with common variants. Researchers continue to identify new loci associated with the risk of SLE, and the new loci account for up to 24% of hereditary SLE cases among previously known loci^6,7^. Although the identification of genes has contributed greatly to the understanding of disease pathogenesis, statistical consistency has been limited^8^. GWAS has focused on common variants, making it difficult to explain the relative impact of rare variants and heritability^9^. Therefore, GWAS has limitations, requiring a new approach to candidate gene identification.

Apoptosis is also known as planned cell death or cell suicide, and is required for maintenance of overall health through removal of abnormal, damaged, or aged cells. Most apoptotic cells are removed by macrophages through non-inflammatory pathways. Apoptosis is triggered by signalling that is recognized by the macrophage through exposure of phosphatidylserine (PS), a phospholipid^10^. Removal of dying cells avoids exposure to autoantigens, and provides powerful anti-inflammatory and immunosuppressive effects that are important in the prevention of autoimmunity induction. The process of identification and removal of dying cells involves many substances, including Tyro-Axl-Mer (TAM) kinase, protein S, and Gas6. Recent research has shown that TAM receptor tyrosine kinases inhibit inflammation by promoting phagocytosis of apoptotic cells, and prevent development of autoimmunity by inducing phagocytosis and stimulating natural killer cell development^11,12^.

Milk fat globule epidermal growth factor 8 (MFGE8) is a glycoprotein found in lacteal glands and milk fat globules. This protein combines with macrophage integrin and PS in dying cells and acts as a bridge between the 2 substances; thus, MFGE8 is also associated with removal of apoptotic cells^13–15^. Adequate amounts of MFGE8 aid in apoptotic cell removal, but excessive amounts competitively inhibit this function. In a mouse model, deficiency of MFGE8 leads to accumulation of apoptotic lymphocytes in lymphatic organs and autoantibody development, resulting in diseases similar to SLE^16^. Therefore, this study aimed to analyse the association of MFGE8 gene polymorphisms with susceptibility and clinical phenotypes in Korean patients with SLE. In addition, differences in MFGE8 protein expression according to genotype were evaluated.

## Results

### Clinical characteristics of subjects

The mean age in 280 patients with SLE was 35.7 ± 7.8 years, and that in 260 healthy subjects (HSs) was 28.1 ± 7.4 years (Supplementary Table 1). Most SLE patients and HSs were female (n = 259, 92.5% vs. n = 241, 92.7%, respectively). There was no statistical difference in age and sex between the groups. Symptoms in SLE patients included oral ulceration (n = 53, 18.9%), skin rash (n = 49, 17.5%), alopecia (n = 40, 14.3%), arthritis (n = 74, 26.4%), and renal disease (n = 57, 20.4%). The average white blood cell count was 5.63 ± 2.52 × 10³/μL, with average erythrocyte sedimentation rate (ESR) of 23.7 ± 21.4 mm/h, C-reactive protein (CRP) level of 1.05 ± 2.9 mg/dL, complement C3 level of 96.5 ± 34.4 mg/dL, and complement C4 level of 21.5 ± 11.0 mg/dL. Anti-dsDNA antibody was positive in 121 patients (43.2%) and the average SLE Disease Activity Index (SLEDAI) score was 4.5 ± 4.5. Glucocorticoids were used by 241 patients (86.1%), with a cumulative dose of 6,739.9 ± 9,622.6 mg prednisolone-equivalent.

### Determination of *MFGE8* genotypes in Koreans

**Figure 1:**
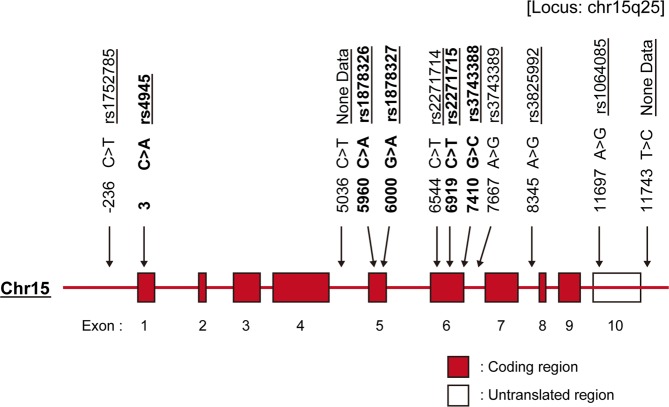
*MFGE8* gene map. The *MFGE8* gene consists of 10 exons separated by 9 introns; Chr15 = chromosome 15; None Data = no SNP data in NCBI database.

DNA sequencing was carried out for the entire *MFGE8* gene in 20 Korean patients with SLE and 20 HSs. We compared DNA sequences and single nucleotide polymorphisms (SNPs) reported in the National Center for Biotechnology Information (NCBI) database, and selected 12 SNPs with at least 5% minor alleles. Two SNPs (5306 C > T, 11743 T > C) were not reported in the NCBI database (Fig. 1). With the addition of 35 patients with SLE and 10 HSs, DNA sequencing was performed for 12 SNPs. Evaluation was performed in a total of 55 SLE patients and 30 HSs with the independent samples *t*-test. We selected 5 target SNPs (rs4945 C/A, rs1878326 C/A, rs1878327 A/G, rs2271715 T/C, and rs3743388C/G) with a P value < 3% (P < 0.03; Supplementary Table 2).

### Association of *MFGE8* polymorphisms with risk of SLE

To identify *MFGE8* genotypes, DNA sequencing was performed in 225 patients with SLE and 230 HSs (total 280 with SLE and 260 HSs). A suitability verification test showed that a genotype frequency of 5 SNPs was needed to maintain hereditary balance according to the Hardy-Weinberg equilibrium (Table 1). Regression analysis showed that the CC genotype in rs2271715 C > A and the GG genotype in rs3743388 G > C were more common in patients with SLE than in HSs (P = 0.036 and P = 0.005, respectively). Linkage disequilibrium assessment of 5 SNPs showed that rs1878326 SNP and rs1878327 SNP were strongly associated (r^2^ = 0.879) (Supplementary Fig. 1). SHEsis software was used to identify 3 haplotypes among 4 SNPs^17^, and the CGCG haplotype showed a statistically significant association with SLE (P = 0.001; Table 2).

**Table 1:** The genotype and allele frequencies of polymorphisms in the *MFGE8* gene.

SNP model	Genotype	SLE (n = 280)	HSs (n = 260)	OR (95% Cl)	p-value
rs4945 C > A					
Additive	CC	235 (83.9)	217 (83.5)	0.954 (0.575–1.583)	0.126
	CA	43 (15.4)	39 (15.0)		
	AA	2 (0.7)	4 (1.5)		
Dominant	CC	235 (83.9)	217 (83.5)	0.966 (0.573–1.628)	0.883
	CA-AA	45 (16.1)	43 (16.5)		
Recessive	CC-CA	280 (100)	256 (98.5)	2.623 (0.419–16.421)	0.053
	AA	0 (0.0)	4 (1.5)		
	MAF	0.084	0.090	1.048 (0.632–1.738)	0.856
	HWE	0.982	0.157		
rs1878326 C > A					
Additive	CC	106 (37.9)	89 (34.2)	1.768 (1.323–2.364)	0.381
	CA	128 (45.7)	128 (49.2)		
	AA	46 (16.4)	43 (16.6)		
Dominant	CC	106 (37.9)	89 (34.2)	1.004 (0.800–1.260)	0.414
	CA-AA	174 (62.1)	171 (65.8)		
Recessive	CC-CA	280 (100)	217 (83.5)	1.082 (0.907–1.290)	0.973
	AA	0 (0.0)	43 (16.5)		
	MAF	0.393	0.412	1.249 (0.839–1.859)	0.274
	HWE	0.485	0.791		
rs1878327 G > A					
Additive	GG	99 (35.4)	84 (32.3)	1.178 (1.275–2.315)	0.455
	GA	139 (49.6)	137 (52.7)		
	AA	42 (15.0)	39 (15.0)		
Dominant	GG	99 (35.4)	84 (32.3)	1.000 (0.789–1.267)	0.479
	GA-AA	176 (67.7)	176 (67.7)		
Recessive	GG-GA	238 (85.0)	221 (85.0)	1.071 (0.895–1.280)	1.000
	AA	42 (15.0)	39 (15.0)		
	MAF	0.398	0.413	1.210 (0.808–1.813)	0.354
	HWE	0.549	0.163		
rs2271715 C > T					
Additive	CC	63 (22.5)	40 (15.4)	3.687 (2.600–5.230)	0.036
	CT	137 (48.9)	135 (51.9)		
	TT	80 (28.6)	85 (32.7)		
Dominant	CC	63 (22.5)	40 (15.4)	1.102 (0.917–1.324)	0.487
	CT-TT	217 (77.5)	220 (84.6)		
Recessive	CC-CT	200 (71.4)	175 (67.3)	1.264 (1.015–1.573)	0.299
	TT	80 (28.6)	85 (32.7)		
	MAF	0.530	0.587	1.637 (1.007–2.660)	0.047
	HWE	0.765	0.255		
rs3743388 G > C					
Additive	GG	68 (24.3)	38 (14.6)	4.253 (2.987–6.056)	0.005
	GC	129 (46.1)	128 (49.2)		
	CC	83 (29.6)	94 (36.2)		
Dominant	GG	68 (24.3)	38 (14.6)	1.159 (0.968–1.388)	0.463
	GC-CC	212 (75.7)	222 (85.4)		
Recessive	GG-GC	197 (70.4)	166 (63.8)	1.369 (1.099–1.705)	0.108
	CC	83 (29.6)	94 (36.2)		
	MAF	0.527	0.608	1.714 (1.049–2.800)	0.032
	HWE	0.203	0.600		

Results are shown as n (%). Each p-value was calculated with additive, dominant and recessive models. Logistic regression analysis was applied to control for age and sex as covariables. MFG-E8: milk fat globule epidermal growth factor 8; SNP: single nucleotide polymorphism; SLE: systemic lupus erythematosus; HSs: healthy subjects; OR: odds ratio; CI: confidence interval; MAF: minor allele frequency; HWE: Hardy-Weinberg equilibrium.

**Table 2:** The haplotype analysis of *MFGE8* gene.

Haplotype	SLE	HSs	Chi-square	p-value	OR (95% CI)
AACG	187 (0.334)	167 (0.321)	0.506	0.477	1.098 (0.848–1.421)
CGCG	52 (0.092)	22 (0.043)	10.790	0.001	2.312 (1.385–3.858)
CGTC	263 (0.469)	268 (0.516)	1.484	0.223	0.858 (0.671–1.098)

Results are shown as n (%). Haplotypes about four single nucleotide polymorphisms were analyzed using SHEsis software^17^. MFG-E8: milk fat globule epidermal growth factor 8; SLE: systemic lupus erythematosus; HSs: healthy subjects; OR: odds ratio; CI: confidence interval.

### Association of *MFGE8* gene polymorphisms with clinical features in patients with SLE

We studied the potential genetic association between *MFGE8* gene polymorphisms and clinical features of SLE (Table 3). In rs4945, the ESR was lower in patients with the CC genotype than in those with CA or AA genotypes (22.6 ± 19.6 mm/h vs. 28.1 ± 29.3 mm/h, P = 0.004). In rs1878326, CRP levels were higher (1.33 ± 3.4 mg/dL vs. 0.52 ± 1.5 mg/dL, P < 0.001) and anti-dsDNA antibody levels were higher (12.6 ± 21.5 IU/mL vs. 8 ± 14.3 IU/mL, P < 0.001) in patients with the CC genotype than in others. In addition, active disease (SLEDAI score >6) was more common in patients with CC genotype (34% vs. 15.5%, P < 0.001), with higher cumulative glucocorticoid doses (8,561 ± 11,239 mg vs. 5,651 ± 8,391 mg prednisolone-equivalent, P = 0.007). In rs1878327, patients with GG genotype had a higher frequency of alopecia (20.2% vs. 11.0%, P = 0.037) and active disease (SLEDAI score > 6) (34.4% vs. 16%, P < 0.001) than other genotypes. The GG genotype also showed higher CRP levels (1.42 ± 4 mg/dL vs. 0.51 ± 1.5 mg/dL, P < 0.001), lower complement C3 levels (92.7 ± 42 mg/dL vs. 96 ± 33.2 mg/dL, P = 0.014), and higher anti-dsDNA antibody levels (12.2 ± 21.3 IU/mL vs. 8.4 ± 14.9 IU/mL, P = 0.004).

**Table 3:** Comparison of the clinical manifestations according to the genotype of *MFGE8* gene in patients with SLE.

Characteristics	rs4945 C > A		rs1878326 C > A		rs1878327 G > A		rs2271715 C > T		rs3743388 G > C	
	CC n = 235 (83.9%)	CA, AA n = 45 (16.1%)	CC n = 106 (37.9%)	CA, AA n = 174 (62.1%)	CC n = 99 (35.4%)	GG, AA n = 181 (64.6%)	CC n = 63 (22.5%)	CT, TT n = 217 (77.5%)	GG n = 99 (35.4%)	GG, CC n = 181 (64.6%)
Alopecia	36 (15.3)	4 (8.9)	20 (18.9)	20 (11.5)	20 (20.2)	20 (11.0)^*^	11 (17.5)	29 (13.4)	12 (17.6)	28 (13.2)
Arthritis	66 (28.0)	8 (17.8)	34 (32.0)	40 (23.0)	30 (30.3)	44 (24.3)	14 (22.3)	60 (27.6)	18 (26.5)	56 (26.4)
Renal disease	45 (19.1)	12 (26.7)	24 (22.6)	33 (19.0)	22 (22.3)	35 (19.3)	23 (36.5)	34 (15.7)^***^	24 (35.3)	33 (15.6)^***^
Platelet, x10³/μL	222.9 ± 83.3	222.3 ± 66.1	211.7 ± 85.7	229.5 ± 77.0	210.1 ± 86.2	229.7 ± 76.9	221.8 ± 70.0	223.0 ± 83.8	215.1 ± 67.6	225.2 ± 84.5
ESR, mm/h	22.6 ± 19.6	28.1 ± 29.3^**^	27.2 ± 23.5	21.2 ± 20.0	26.5 ± 23.2	21.8 ± 20.4	26.0 ± 21.2	22.8 ± 21.6	25.8 ± 22.1	22.8 ± 21.3
CRP, mg/dL	0.84 ± 2.76	0.77 ± 2.05	1.33 ± 3.4	0.52 ± 1.5^***^	1.42 ± 4.0	0.51 ± 1.5^***^	0.80 ± 2.1	0.84 ± 2.8	1.12 ± 3.0	0.74 ± 2.5
Complement3, mg/dL	95.9 ± 37.4	89.2 ± 31.2	92.2 ± 40.4	95.7 ± 34.0	92.7 ± 42.0	96.0 ± 33.2^*^	85.9 ± 34.0	97.4 ± 36.9	86.8 ± 31.4	97.3 ± 37.6
Complement4, mg/dL	20.7 ± 11.3	21.6 ± 12.8	19.3 ± 12.6	21.7 ± 10.8	19.0 ± 12.7	21.8 ± 10.8	18.0 ± 9.6	21.6 ± 12.0	19.4 ± 10.2	21.3 ± 12.0
Anti-dsDNA Ab, IU/mL	10.2 ± 18.4	7.1 ± 11.2	12.6 ± 21.5	8.0 ± 14.3^***^	12.2 ± 21.3	8.4 ± 14.9	11.4 ± 20.5	9.3 ± 16.5	11.4 ± 21.4	9.2 ± 16.1^*^
SLEDAI, > 6	54 (23.0)	9 (20.0)	36 (34.0)	27 (15.5)^***^	34 (34.4)	29 (16.0)^***^	15 (23.8)	48 (22.1)	18 (26.5)	45 (21.2)
Glucocorticoid cumulative dose, g	7.0 ± 9.8	5.3 ± 8.7	8.6 ± 11.2	5.7 ± 8.4^**^	8.0 ± 10.9	6.0 ± 8.8	10.0 ± 13.2	5.8 ± 8.1^***^	11.0 ± 13.1	5.4 ± 7.8^***^
Azathioprine	37 (15.7)	9 (20.0)	14 (13.2)	32 (18.4)	12 (12.1)	34 (18.8)	11 (17.5)	35 (16.1)	13 (19.1)	33 (15.6)
Cyclophosphamide	20 (8.5)	3 (6.7)	9 (8.5)	14 (8.0)	6 (6.1)	17 (9.4)	9 (14.3)	14 (6.5)^*^	11 (16.2)	12 (5.7)^**^
Mycophenolate mofetil	23 (9.8)	4 (8.9)	13 (12.3)	14 (8.0)	9 (9.0)	18 (9.9)	12 (19.0)	15 (6.9)^*^	13 (19.1	14 (6.6)^**^
Methotrexate	39 (16.6)	6 (13.4)	21 (19.8)	24 (13.8)	18 (18.2)	27 (14.9)	8 (12.7)	37 (17.0)	8 (11.8)	37 (17.5)

Results are shown as n (%) or mean ± SD. Each p value calculated using linear regression analysis and Mann-Whitney U test. Values p < 0.05 were considered statistically significant. *p < 0.05, **p < 0.01, ***p < 0.001. MFGE8: milk fat globule epidermal growth factor 8; SLE: systemic lupus erythematosus; ESR: erythrocyte sedimentation rate; CRP: C-reactive protein; dsDNA: double strand deoxynucleic acid; Ab: antibody; SLEDAI: systemic lupus erythematosus disease activity index; SD: standard deviation.

In rs2271715, renal disease was more common in patients with CC genotype (36.5% vs. 15.7%, P = 0.001) than in other genotypes, with higher cumulative glucocorticoid doses (10,129 ± 13,242 mg vs. 5,767 ± 8,107 mg prednisolone-equivalent, P < 0.001). Patients with CC genotype more commonly took cyclophosphamide (14.3% vs. 6.5%, P = 0.028) and mycophenolate mofetil (MMF) (19.0% vs. 6.9%, P = 0.002).

In rs3743388, anti-dsDNA antibody levels were higher in the GG genotype (11.4 ± 21.4 IU/mL vs. 9.2 ± 16.1 IU/mL, P = 0.023). Patients with the GG genotype also had a higher rate of renal disease (35.3% vs. 15.6%, P = 0.001), with higher cumulative glucocorticoid doses (10,982 ± 13,141 mg vs. 5,393 ± 7,793 mg prednisolone-equivalent, P < 0.001). Cyclophosphamide and MMF were more frequently administered in patients with GG genotype (16.2% vs. 5.7%, P = 0.004, and 19.1% vs. 6.6%, P = 0.001, respectively).

### Analysis of MFGE8 protein expression in patients with SLE

**Figure 2:**
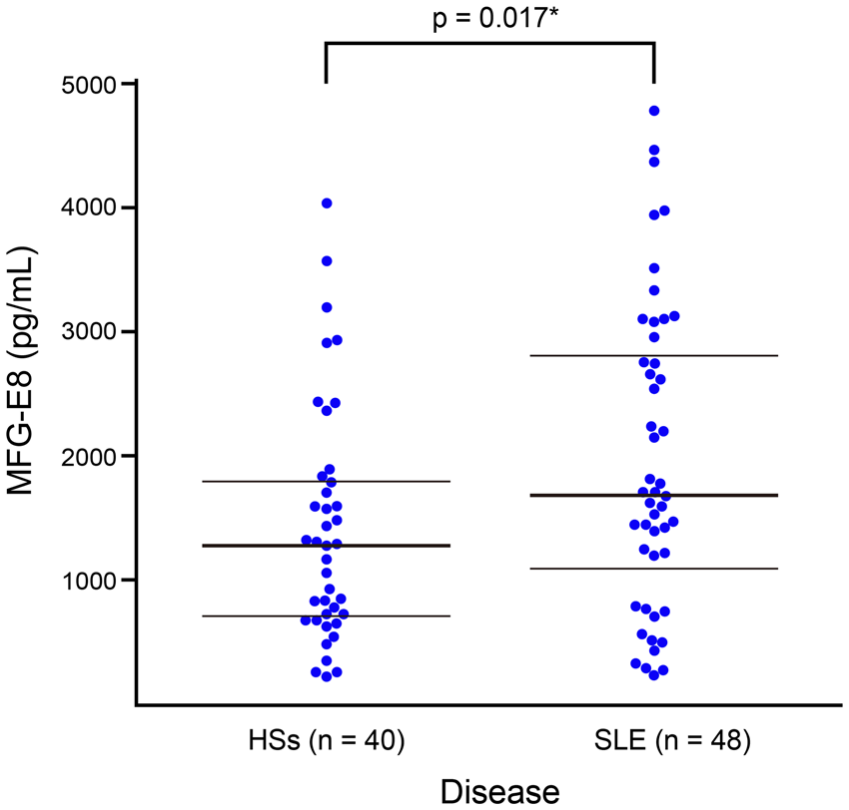
Serum MFGE8 concentration in patients with systemic lupus erythematosus (SLE) and healthy subjects (HSs). Mean age of SLE patients was 34.8 ± 8 years and 87.5% were women, compared with 33 ± 7 years and 87.5% in HSs. Mean serum MFGE8 concentration was 2,030.6 ± 2,308.3 pg/mL in patients with SLE, compared with 1,433 ± 946.3 pg/mL in HSs; P = 0.017.

MFGE8 protein expression was examined in 48 SLE patients with SLEDAI scores >6 and 40 age-and sex-matched HSs (Supplementary Table 3). The serum level of MFGE8 was higher in patients with SLE than in HSs (2,030.6 ± 2,308.3 pg/mL vs. 1,433 ± 946.3 pg/mL, P = 0.017) (Fig. 2).

### Correlation between *MFGE8* gene SNP and protein expression

The level of MFGE8 protein was increased significantly in SLE patients with rs4945 CA or AA genotype than in SLE patients with rs4945 CC genotype (5,136.1 ± 2,140.5 pg/mL vs. 1841.8 ± 1,168.6 pg/mL, P < 0.001; Table 4). In addition, NCs with rs2271715 CC genotype had significantly higher MFGE8 protein levels (1,571.6 ± 461.1 pg/mL vs. 1407.7 ± 1,022.9 pg/mL, P = 0.037). When comparing the MFGE8 protein level according to genotype, SLE patients with rs4945 CA or AA genotype had higher levels than HSs with the same genotype (2454.8 ± 480.4 pg/mL, P = 0.035). Furthermore, in the rs2271715 CC genotype, patients with SLE showed higher MFGE8 protein levels than those in HSs (2460,2 ± 1,158.3 pg/mL vs. 1,571.6 ± 461.1 pg/mL, P = 0.004).

**Table 4:** Serum MFG-E8 concentration according to the genotype of *MFGE8* gene in 48 patients with SLE and 40 healthy subjects.

SNP Genotype	SLE (n = 48)		HSs (n = 40)		SLE vs. HSs
	n (%)	MFGE8 (pg/mL) mean ± SD	n (%)	MFGE8 (pg/mL) mean ± SD	p-value
rs4945					
CC	42 (87.5)	1,841.8 ± 1,168.6	33 (82.5)	1,470.9 ± 1,019.6	0.157
CA, AA	6 (12.5)	5,136.1 ± 2,140.5	7 (17.5)	1,254.8 ± 480.4	0.035
rs1878326					
CC	28 (58.3)	2,615.1 ± 2,843.5	16 (40.0)	1,746.4 ± 1,095.4	0.306
CA, AA	20 (41.7)	1,745.5 ± 1,108.0	24 (60.0)	1,224.1 ± 788.6	0.064
rs1878327					
GG	28 (58.3)	2,615.1 ± 2,843.5	17 (42.5)	1,660.0 ± 1,119.0	0.190
GA, AA	20 (41.7)	1,747.5 ± 1,108.0	23 (57.5)	1,265.4 ± 779.4	0.091
rs2271715					
CC	8 (16.7)	2,460.2 ± 1,158.3	7 (17.5)	1,571.6 ± 461.1	0.004
CT, TT	40 (83.3)	2,212.3 ± 2,484.0	33 (82.5)	1,403.7 ± 1,022.9	0.079
rs3743388					
GG	9 (18.7)	2,579.6 ± 1,141.1	6 (15.0)	1,201.8 ± 557.1	0.112
GC, CC	39 (81.3)	2,178.4 ± 2,507.0	34 (85.5)	1,473.8 ± 999	0.139

Each p value calculated using linear regression analysis and Mann-Whitney U test. MFG-E8: milk fat globule epidermal growth factor 8; SNP: single nucleotide polymorphism; SLE: systemic lupus erythematosus; HSs: healthy subjects; SD: standard deviation.

## Discussion

This study identified 5 SNPs by searching the entire *MFGE8* genome in Korean patients with SLE. The CC genotype of rs2271715 and the GG genotype of rs3743388 were more common in patients with SLE than in HSs. In haplotype analysis, the CGCG haplotype was found to have a 2.3-fold increased risk of SLE. In addition, these SNPs were associated with several disease activity markers and renal disease in SLE. Furthermore, the expression level of MFGE8 protein in SLE patients was significantly higher than in HSs with the same genotype.

The Human Genome Project and International Human HapMap Project have provided reference information on genetic variations associated with complex diseases, and advances in microarray technology have now allowed large numbers of SNP markers to be screened at once^18–20^. However, GWAS based on a common variant hypothesis have generated controversy^21^. Although new genetic markers have been discovered through use of GWAS, various indicators offer little explanation. Since GWAS cannot account for the association between a signalling pathway and a gene, the findings cannot be used to identify and prevent the causes of a disease^22^. Therefore, a candidate gene analysis is required for precise identification of an association.

MFGE8 is a bridging molecule interposed between apoptotic cells and phagocytes that plays an important role in the clearance of dying cells. The level of MFGE8 protein is thought to be associated with the pathogenesis of SLE. Research on MFGE8 has been reported from Japan, Taiwan, and the USA^23–27^; however, no prior study has correlated *MFGE8* polymorphism with function. Thus, this study evaluated the association between *MFGE8* polymorphisms and SLE based on MFGE8 protein levels in Korean patients with SLE.

First, DNA sequencing was performed on the entire *MFGE8* gene to compare SNPs reported to the NCBI with 5 verified SNPs showing minor alleles of 3% or more (rs4945, rs1878326, rs1878327, rs2271715, and rs3743388). Among these, rs4945 has also been studied in Taiwanese patients with SLE. When only genotypes were compared, the rs2271715 C allele and rs3743388 G allele were more common in patients with SLE than in HSs. According to data reported to the NCBI, the rs2271715 T allele and rs3743388 C allele were more common in Europeans, Chinese, Japanese, and Koreans. Thus, it was notable that the rs2271715 C allele and rs3743388 G allele frequencies were higher in patients with SLE. An examination of 3 haplotypes among 4 SNPs in the CGCG haplotype showed statistically significant differences between patients with SLE and HSs, (P = 0.001), with a 2.3-fold increased risk of developing SLE. An analysis of the correlations between clinical characteristics in SLE and MFGE8 SNP showed that rs4945 showed an association with ESR, rs1878326 showed an association with CRP, anti-dsDNA antibody, SLEDAI score, and glucocorticoid dose, and rs1878327 showed an association with alopecia, CRP, complement C3, anti-dsDNA, and SLEDAI score. rs2271715 and rs3743388 showed associations with kidney disease, glucocorticoid dose, and use of cyclophosphamide and MMF. In addition, rs3743388 SNP showed an association with anti-dsDNA antibody. These results suggest that these 5 SNPs are associated not only with the occurrence of SLE but also with clinical manifestations.

There was a report that the level of MFGE8 in the blood of patients with type 2 diabetes was higher than HSs^24^. Reduction of expression of MFGE8 in diabetic mouse models alleviated atherosclerosis, a major complication of diabetes. Therefore, the expression level of MFGE8 is a very important factor in its function, and can be said to be related to the pathogenesis of autoimmune diseases including SLE. In addition, serum MFGE8 levels in Japanese patients with SLE were significantly higher than those in healthy individuals^23,28^. Moreover, the medical treatment of SLE resulted in significantly lower levels of MFGE8. In another study, MFGE8 overproduction in patients with SLE protected against lupus-related damage by reducing neutrophil migration and the development of neutrophil extracellular traps (NETosis)^29^.

We found that levels of serum MFGE8 were higher in patients with SLE than in HSs. This is consistent with the results of prior studies. Additionally, we examined the correlation between *MFGE8* genotype and protein level. The level of MFGE8 protein was significantly higher in SLE patients with the rs2271715 CC genotype than in HSs with the same genotype. This suggests that the increased level of MFGE8 in SLE patients with the increased-risk genotype of rs2271715 is involved in the pathogenesis of SLE. In addition, high MFGE8 levels may cause competitive inhibition in the phagocytosis of apoptotic cells. This can result in incomplete elimination of apoptotic cells, in turn resulting in autoantigen exposure to immune cells in SLE. Unravelling the mechanisms of MFGE8 overexpression-driven SLE may increase knowledge about autoimmune disease, but additional studies are required.

Our results have shown that MFGE8 rs2271715 and rs3743388 SNP not only increase the risk of SLE by increasing MFGE8 protein but also influence SLE disease activity. Moreover, rs4945, rs1878326, and rs1878327 can be used as biomarkers of SLE disease activity. In addition, the level of MFGE8 protein was higher in patients with SLE than in HSs. The strength of our study is the inclusion of clinical manifestations of SLE and the determination of serum MFGE8 levels.

In relation to lupus nephritis and MFGE8 protein, it was reported that MFG-E8 genetic polymorphism studies showed glomerulonephritis in both MFGE8 deficient mice and SLE patients, but did not show significant differences in MFGE8 genotype distributions between SLE patients with or without lupus nephritis^26^. In our study showed that rs2271715 and rs3743388 showed associations with kidney disease and use of cyclophosphamide and MMF.

In conclusion, *MFGE8* genetic polymorphisms are associated not only with susceptibility to SLE but also with disease activity through their influence on gene expression.

## Methods

### SLE patients and healthy subjects

The study included 280 patients with SLE and 260 HSs. All patients satisfied at least 4 of the revised American College of Rheumatology (1997) criteria for the classification of SLE^30^. This study was approved by the Institutional Review Board of Ajou University Hospital (AJIRB-BMR-GEN-15-237), and informed consents were given by all subjects. All procedures for this study were carried out in accordance with the approved guidelines. The clinical features, serum test results, and medication status of patients were collected and registered in a database. Blood samples were collected from patients with SLE and HSs and immediately stored at −70 °C.

### Genomic DNA extraction

Genomic DNA was extracted from whole blood of patients with SLE and HSs using a Total DNA Extraction Mini kit (Intron Biotechnology, Seoul, Korea), according to the manufacturer’s instructions. The eluted genomic DNA samples were quantified individually using a spectrophotometer (Nanodrop Lite; Thermo, Waltham, MA, USA).

### Identification of single nucleotide polymorphisms in *MFGE8* genes

SNPs were genotyped in 3 steps. First, SNPs were identified as DNA sequences with at least 5% minor allele frequencies, compared to nucleotide sequences. The presence of previously reported SNPs was confirmed using dbSNP (http://www.ncbi.nlm.nih.gov/projects/SNP). We then designed forward and reverse primers (18 each) of the *MFGE8* gene using the nucleotide sequence reported in the NCBI (http://www.ncbi.nlm.nih.gov) to determine the entire single nucleotide polymorphism of the *MFGE8* gene (Supplementary Table 4). Polymerase chain reaction analysis was performed on 20 genomic DNA samples from patients with SLE and HSs using these primers. We then selected 12 SNPs with at least 5% minor allele frequencies. Next, DNA sequencing of the *MFGE8* gene was performed (Cosmogenetech, Seoul, Korea) using 36 primers. The results of the analysis were confirmed with a sequencer program (Gene Codes Corporation, Ann Arbor, MI, USA), and the nucleotide sequences of 55 patients with SLE and 30 HSs were compared with an independent samples *t*-test. Finally, we selected 5 target SNPs (rs4945 C/A, rs1878326 C/A, rs1878327 A/G, rs2271715 T/C, and rs3743388C/G) with a P value < 3%.

### Enzyme-linked immunosorbent assay

The serum concentration of MFGE8 was analysed using sandwich ELISA (Human MFGE8 Quantikine ELISA Kit; R&D Systems, Minneapolis, MN, USA) according to the manufacturers’ protocols. The absorbance was measured at 450 nm on a Microplate Absorbance Spectrophotometer (Bio-Rad Laboratories, Hercules, CA, USA), and the data were analysed against a standard curve using Microplate Manager^®^ Software (Bio-Rad Laboratories).

### Statistical analysis

The SNP model to be analysed was in agreement with the inherited form, regardless of statistical significance, using 3 models: additive, dominant, and recessive. Next, each SNP was converted into these 3 models and an independent samples *t*-test was performed to confirm the significance of the association between the SLE and HSs. In addition, multiple linear regression analysis, the Mann-Whitney U test, and analysis of variance were performed using various dominant models of SNPs, clinical features, serum test results, and medication history. Differences in MFGE8 protein levels between patients with SLE and HSs were determined with an independent samples *t*-test. After evaluation of correlations between MFGE8 SNP and expression levels of MFGE8 protein, differences in protein levels between the SLE and HSs with same SNP were analysed using the Mann-Whitney U test and multiple linear regression analysis. P values < 0.05 were considered statistically significant. Statistical analysis was performed using IBM SPSS Statistics 23.0 software (IBM Corp., Armonk, NY, USA) and the R version 3.2.5 program (The R Foundation for Statistical Computing Platform, Vienna, Austria).

## Supplementary information

Supplementary Tables & Figure
