# Supplementary material for: Polymorphisms of MFGE8 are associated with susceptibility and clinical manifestations through gene expression modulation in Koreans with systemic lupus erythematosus
Source: Sci Rep. 2019 Dec 6;9:18565. doi: 10.1038/s41598-019-55061-6 (PMC6897915; doi:10.1038/s41598-019-55061-6)
Supplement: Supplementary file 1 — Supplementary Tables & Figure [file 41598_2019_55061_MOESM1_ESM.docx]

**Polymorphisms of *MFGE8* are associated with susceptibility and clinical manifestations through gene expression modulation in Koreans with systemic lupus erythematosus**

Wook-Young Baek, PhD ^#^, Ji-Min Woo, MS ^#^, Hyoun-Ah Kim, MD, PhD, Ju-Yang Jung, MD, Chang-Hee Suh, MD, PhD ^*^

Department of Rheumatology, Ajou University School of Medicine, 164 Worldcup-ro, Yeongtong-gu, Suwon 16499, Korea

**Supplementary Table S1.** Clinical characteristics of the patients with SLE and healthy subjects.

| **Characteristics** | **SLE (n = 280)** | **HS (n = 260)** | **p-value** |
| --- | --- | --- | --- |
| Age, years | 35.7 ± 7.8 | 28.1 ± 7.4 | 0.651 |
| Sex, F:M | 92.5:7.5 | 92.7:7.3 | 0.865 |
| Manifestations |  |  |  |
| Oral ulcer | 53 (18.9) |  |  |
| Skin rash | 49 (17.5) |  |  |
| Alopecia | 40 (14.3) |  |  |
| Arthritis | 74 (26.4) |  |  |
| Renal disease | 57 (20.4) |  |  |
| Laboratory data |  |  |  |
| WBC, x10³/μL | 5.63 ± 2.52 |  |  |
| Lymphocyte, x10³/μL | 1.40 ± 0.64 |  |  |
| Hemoglobin, g/dL | 12.1 ± 1.5 |  |  |
| Platelet, x10³/μL | 224.4 ± 78.6 |  |  |
| ESR, mm/h | 23.7 ± 21.4 |  |  |
| CRP, mg/dL | 1.05 ± 2.9 |  |  |
| Complement3, mg/dL | 96.5 ± 34.4 |  |  |
| Complement4, mg/dL | 21.5 ± 11.0 |  |  |
| Anti-dsDNA Ab positivity | 121 (43.2) |  |  |
| SLEDAI | 4.5 ± 4.5 |  |  |
| Medication |  |  |  |
| Glucocorticoid | 241 (86.1) |  |  |
| Glucocorticoid cumulative dose, mg | 6,739.9 ± 9,622.6 |  |  |
| Azathioprine | 48 (17.1) |  |  |
| Cyclophosphamide | 25 (8.9) |  |  |
| Mycophenolate mofetil | 29 (10.4) |  |  |
| Methotrexate | 47 (16.8) |  |  |

Results are shown as n (%) or mean ± SD. SLE: systemic lupus erythematosus; HS: healthy subject; WBC: white blood cell; ESR: erythrocyte sedimentation rate; CRP: C-reactive protein; dsDNA: double strand deoxynucleic acid; Ab: antibody; SLEDAI: systemic lupus erythematosus disease activity index; SD: standard deviation.

**Supplementary Table S2.** Single nucleotide polymorphisms within the *MFGE8* gene identified in 55patients with SLE and 30 healthy subjects.

| **SNP** | **Heterozygosity** | **Function** | **dbSNP allele** |
| --- | --- | --- | --- |
| rs4945 | 0.2676 | Missense mutation | C/A |
| rs1878326 | 0.3616 | Missense mutation | C/A |
| rs1878327 | 0.3710 | Synonymous codon | G/A |
| rs2271715 | 0.3644 | Intron variant | C/T |
| rs3743388 | 0.4427 | Intron variant | C/G |

MFGE8: milk fat globule epidermal growth factor 8; SLE: systemic lupus erythematosus; SNP: single nucleotide polymorphisms; dbSNP: Single nucleotide polymorphism database. SNP selected by direct sequencing of MFG-E8 gene.

**Supplementary Table S3.** Clinical characteristics of the patients with SLE and healthy subjects.

| **Characteristics** | **SLE (n = 48)** | **HS (n = 40)** | **SLE vs. HS p-value** |
| --- | --- | --- | --- |
| Age, years | 34.8 ± 8.0 | 33.1 ± 7.0 | 0.308 |
| Sex, F:M | 92.5:7.5 | 92.5:7.5 | 1.000 |
| Oral ulcer | 14 (18.9) |  |  |
| Skin rash | 14 (18.9) |  |  |
| Alopecia | 17 (35.4) |  |  |
| Arthritis | 29 (60.4) |  |  |
| Renal disease | 29 (60.4) |  |  |
| Laboratory data |  |  |  |
| WBC, x10³/μL | 6.0 ± 3.1 |  |  |
| Lymphocyte, x10³/μL | 1.1 ± 0.7 |  |  |
| Hemoglobin, g/dL | 11.5 ± 1.7 |  |  |
| Platelet, x10³/μL | 200.8 ± 93.1 |  |  |
| ESR, mm/h | 33.5 ± 26.8 |  |  |
| CRP, mg/dL | 1.7 ± 3.1 |  |  |
| Complement3, mg/dL | 87.2 ± 48.2 |  |  |
| Complement4, mg/dL | 17.0 ± 14.0 |  |  |
| Anti-dsDNA Ab positivity | 27 (56.2) |  |  |
| SLEDAI | 9.9 ± 5.5 |  |  |
| Medication |  |  |  |
| Glucocorticoid | 38 (79.1) |  |  |
| Glucocorticoid cumulative dose, mg | 4,449.5 ± 8,353.6 |  |  |
| Azathioprine | 3 (6.3) |  |  |
| Cyclophosphamide | 1 (2.0) |  |  |
| Mycophenolate mofetil | 1 (2.0) |  |  |
| Methotrexate | 5 (10.4) |  |  |

Results are shown as n (%) or mean ± SD. SLE: systemic lupus erythematosus; healthy subject: healthy subject; WBC: white blood cell; ESR: erythrocyte sedimentation rate; CRP: C-reactive protein; dsDNA: double strand deoxynucleic acid; Ab: antibody; SLEDAI: systemic lupus erythematosus disease activity index; SD: standards deviation.

**Supplementary Table S4.** Primer design of *MFGE8* gene for polymerase chain reaction.

| **Primer name** | **Design** | **Tm** | **Size** |
| --- | --- | --- | --- |
| P01F | TTTGCACATGGGAGTTTCTG | 59.69 | 908 bp |
| P01R | GAGTCCCAGCCCAAAAGC | 60.75 | 908 bp |
| P02F | CCTCCACCTCCACTGTTGAC | 60.56 | 980 bp |
| P02R | TGGGACTTCAGAGTCCTGCT | 59.99 | 980 bp |
| P03F | CCCCTTTCTACTCTTCCCTGT | 58.72 | 986 bp |
| P03R | CCTGTCCTCAATCCCTTGAC | 59.51 | 986 bp |
| P04F | AGCCCTTCTGGGATGCTAAT | 60.06 | 958 bp |
| P04R | CTGACTCAGGAACAGCACGA | 60.00 | 958 bp |
| P05F | CGCTGCATCTGATTTCTCTG | 59.70 | 992 bp |
| P05R | CTCACTCCTCTGCATCAGCA | 60.18 | 992 bp |
| P06F | GGGAAAAGGGTCCTGTTGTT | 60.20 | 983 bp |
| P06R | ATTCCCACTCAGGCACACTC | 60.12 | 983 bp |
| P07F | GGGTTGCTAAGGCAATCAGA | 60.21 | 988 bp |
| P07R | ATATAGGGCATCTGGCAGCA | 60.59 | 988 bp |
| P08F | AGGGGACACTGAGGAAGAGG | 60.64 | 983 bp |
| P08R | AGCAGACCCATCCCTTCAC | 60.06 | 983 bp |
| P09F | ACCCCTCCTCTGTTTTGCTT | 60.64 | 950 bp |
| P09R | CCCTGGGGTTACCTCATCTT | 60.06 | 950 bp |
| P10F | ACCCCTCCTCTGTTTTGCTT | 60.11 | 986 bp |
| P10R | CCCTGGGGTTACCTCATCTT | 60.18 | 986 bp |
| P11F | CTCTGCTGGCTCTTCCAAGT | 59.75 | 934 bp |
| P11R | GGCTCTGCAGAAATCTGTCC | 59.96 | 934 bp |
| P12F | GCAGACATATGGTGTCTTTGG | 58.09 | 996 bp |
| P12R | AGGACTGTCAGCTCCAGCAC | 60.62 | 996 bp |
| P13F | GGGAGACTGAGAGCCAGAGA | 59.66 | 955 bp |
| P13R | GATGAAGGCCTGCCTAGTGA | 60.36 | 955 bp |
| P14F | CCCCAGGAGTTGTCTGATTT | 58.99 | 970 bp |
| P14R | CCATATCCCAAGAAGGCTGA | 60.03 | 970 bp |
| P15F | ACAAGCAGGGCAACTTCAAC | 60.30 | 986 bp |
| P15R | CCCCAACATCCAGGTGTAAA | 60.61 | 986 bp |
| P16F | TAGATGAGGAAGCCGAGAGC | 59.68 | 978 bp |
| P16R | GGACAGAGCCAAAGTTACGG | 59.73 | 978 bp |
| P17F | CTCCTCGAAGGAGGTGACAG | 59.98 | 844 bp |
| P17R | ACGGGCAAGAGGCTGTTAT | 60.00 | 844 bp |
| P18F | CTCCTAGCCCCTCTCTCACA | 60.00 | 952 bp |
| P18R | CTGACCAGGCAGCAGTTGTA | 55.00 | 952 bp |

MFGE8: milk fat globule epidermal growth factor 8; Tm: melting temperature; F: forward; R: reverse.


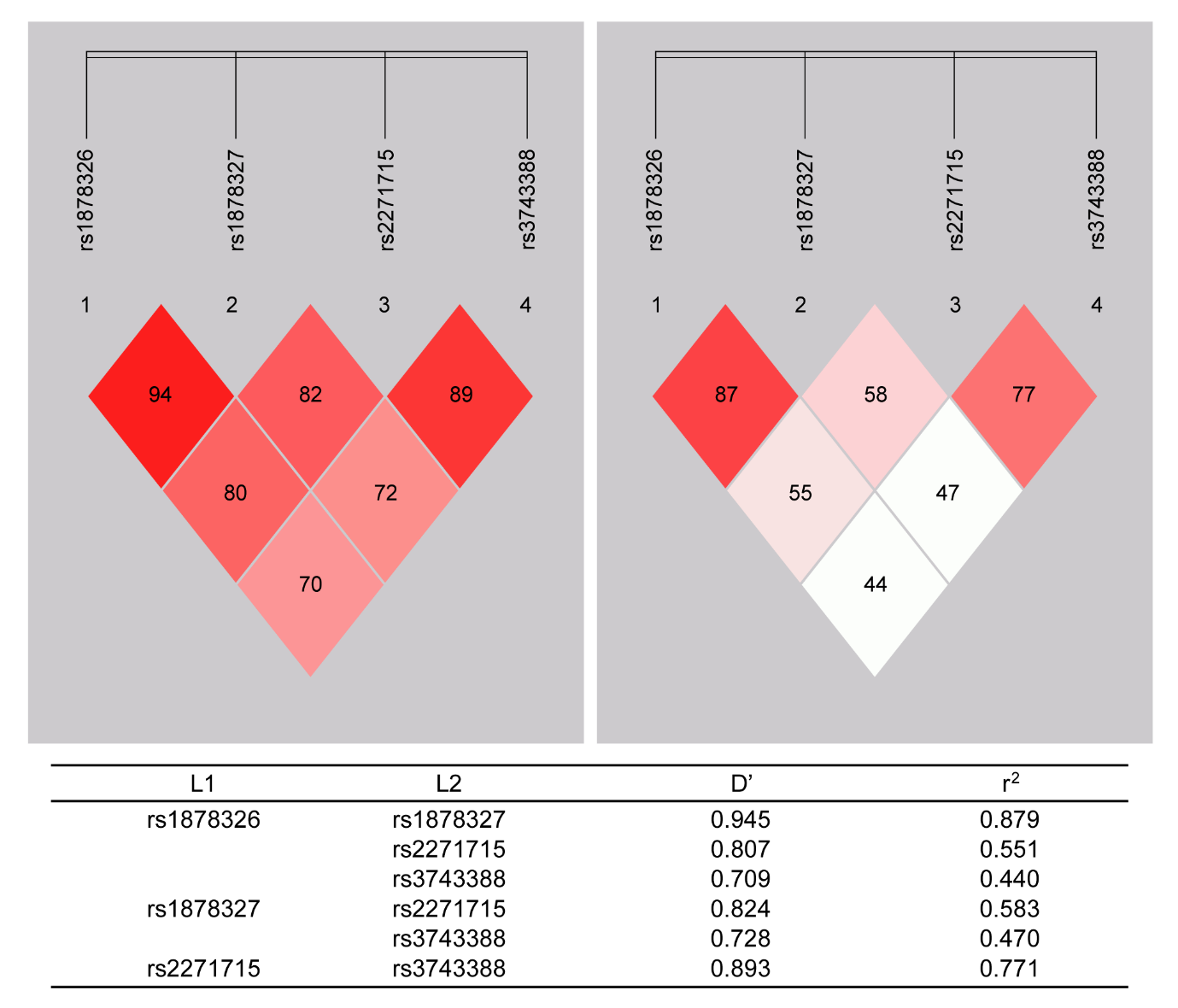


**Supplementary Figure S1.** The linkage disequilibrium (LD) coefficients (D' and r^2^) among four single nucleotide polymorphisms in MFG-E8 gene. MFGE8: milk fat globule epidermal growth factor 8.
